# Anterior cingulate cortex mediates the relationship between O3PUFAs and executive functions in APOE e4 carriers

**DOI:** 10.3389/fnagi.2015.00087

**Published:** 2015-05-21

**Authors:** Marta K. Zamroziewicz, Erick J. Paul, Rachael D. Rubin, Aron K. Barbey

**Affiliations:** ^1^Decision Neuroscience Laboratory, University of IllinoisUrbana, IL, USA; ^2^Beckman Institute for Advanced Science and Technology, University of IllinoisUrbana, IL, USA; ^3^Neuroscience Program, University of IllinoisChampaign, IL, USA; ^4^Carle Neuroscience Institute, Carle Foundation HospitalUrbana, IL, USA; ^5^Department of Speech and Hearing Science, University of IllinoisChampaign, IL, USA; ^6^Department of Internal Medicine, University of IllinoisChampaign, IL, USA; ^7^Institute for Genomic Biology, University of IllinoisChampaign, IL, USA; ^8^Department of Psychology, University of IllinoisChampaign, IL, USA

**Keywords:** omega-3 fatty acids, anterior cingulate cortex, executive function, cognitive aging, nutritional neuroscience

## Abstract

**Introduction**: Although diet has a substantial influence on the aging brain, the relationship between biomarkers of diet and aspects of brain health remains unclear. This study examines the neural mechanisms that mediate the relationship between omega-3 polyunsaturated fatty acids (O3PUFAs) and executive functions in at-risk (APOE e4 carriers), cognitively intact older adults. We hypothesized that higher levels of O3PUFAs are associated with better performance in a particular component of the executive functions, namely cognitive flexibility, and that this relationship is mediated by gray matter volume of a specific region thought to be important for cognitive flexibility, the anterior cingulate cortex.

**Methods**: We examined 40 cognitively intact adults between the ages of 65 and 75 with the APOE e4 polymorphism to investigate the relationship between biomarkers of O3PUFAs, tests of cognitive flexibility (measured by the Delis-Kaplan Executive Function System Trail Making Test), and gray matter volume within regions of the prefrontal cortex (PFC).

**Results**: A mediation analysis revealed that gray matter volume within the left rostral anterior cingulate cortex partially mediates the relationship between O3PUFA biomarkers and cognitive flexibility.

**Conclusion**: These results suggest that the anterior cingulate cortex acts as a mediator of the relationship between O3PUFAs and cognitive flexibility in cognitively intact adults thought to be at risk for cognitive decline. Through their link to executive functions and neuronal measures of PFC volume, O3PUFAs show potential as a nutritional therapy to prevent dysfunction in the aging brain.

## Introduction

As the aged population expands, the economic burden of care and treatment of those with age-related health disorders also increases. The current and projected prevalence of Alzheimer’s disease, for example, suggests an increase in the United States from 5.1 to 13.2 million by 2050 with healthcare expenditures related to this growth expected to surpass 1 trillion dollars (Alzheimer’s Association, 2013). Therefore a successful strategy to promote healthy brain aging is of great interest to public health efforts and the United States economy. Diet and the many bioactive substances present in food represent a novel target for intervention that may promote healthy brain aging. Defining the precise mechanisms through which diet may impact brain health is a first step to developing successful dietary strategies against brain aging.

Accumulating evidence indicates that omega-3 polyunsaturated fatty acids (O3PUFAs) have a beneficial effect on cognitive aging. These long-chain polyunsaturated fatty acids serve as structural components of neuronal membranes and may have neuroprotective properties through anti-inflammatory, antioxidant, and energy metabolism pathways (Cunnane et al., 2009). However, the core brain regions that O3PUFAs may act upon are unknown. This study aims to investigate specific and sensitive neural mechanisms that mediate the beneficial effect of O3PUFAs on cognitive aging, and in particular, the brain regions that underlie the relationship between O3PUFAs and cognitive flexibility, a component of the executive functions. This investigation focuses on carriers of the APOE e4 allele to evaluate these relationships in cognitively intact aging individuals thought to be at risk for cognitive decline (Jorm et al., 2007; Kozauer et al., 2008; Wisdom et al., 2011; Bell et al., 2012; Schiepers et al., 2012; Davies et al., 2014).

O3PUFAs have been to linked to superior cognitive performance, primarily in tasks that require executive functions (Beydoun et al., 2007; Witte et al., 2013; Bauer et al., 2014). Executive functions have been defined in many ways, but traditionally consist of planning and execution of goal-directed behaviors, abstract reasoning, and judgment (Stuss and Alexander, 2000). More recently, executive functions have been defined as the efficiency with which an individual applies his or her knowledge to cope with everyday life (Princiotta and Devries, 2014). The presence of executive dysfunction without measureable deficits in general cognition may represent the continuum of normal aging or a preclinical stage of dementia (Johnson et al., 2007). In particular, O3PUFAs have been directly linked to better performance on tasks that entail a particular component of the executive functions, namely cognitive flexibility (Bowman et al., 2013; Johnston et al., 2013). Cognitive flexibility refers to the ability to adjust to new demands or rules, and can be measured using task switching paradigms (Diamond, 2013).

Executive functions are implemented in the prefrontal cortex (PFC), and specific aspects of executive functions may be localized to particular sub-regions within the PFC (Barbey et al., 2012, 2013a,b,c, 2014a,b). In general, higher gray matter volume in the PFC has been associated with better performance on tasks that elicit executive functions (Kochunov et al., 2009; Burzynska et al., 2012; Tu et al., 2012). More specifically, and of interest in this study, one of the anatomically distinct sub-regions of the ventromedial PFC known as the anterior cingulate is thought to underlie aspects of cognition known as attention, cognitive control, working memory, set maintenance, and goal directed behavior (Barbey et al., 2011; Nee et al., 2011). In fact, higher gray matter volumes in the anterior cingulate cortex have been related to the cognitive flexibility component of the executive functions, as evidenced by performance on measures of task switching (Huster et al., 2009; Nee et al., 2011).

Given that O3PUFAs impact cognitive flexibility and that cognitive flexibility seems reliant upon the anterior cingulate cortex, we examined the role of regions within the PFC in mediating the relationship between O3PUFAs, as measured by blood biomarkers, and this particular component of the executive functions in cognitively intact aging individuals at risk for cognitive decline. In light of existing evidence, we predict that gray matter volume of the anterior cingulate would mediate the beneficial effect of O3PUFAs on cognitive flexibility.

## Materials and Methods

### Participants

This cross-sectional study enrolled 95 elderly adults from Carle Foundation Hospital, a local and readily available cohort of well-characterized elderly adults. No participants were cognitively impaired, as defined by a score of lower than 26 on the Mini-Mental State Examination. Participants with a diagnosis of mild cognitive impairment, dementia, psychiatric illness within the last 3 years, stroke within the past 12 months, and cancer within the last 3 years were excluded. Participants were also excluded for current chemotherapy or radiation, an inability to complete study activities, prior involvement in cognitive training or dietary intervention studies, and contraindications for MRI. Of these 95 participants, only a subset (*n* = 52) underwent genotyping of APOE alleles. Of this subset, 40 participants had the APOE e4 allele whereas 12 participants did not carry the APOE e4 allele. Our hypothesis focuses on APOE e4 carriers who may be at higher risk for cognitive decline and our sample does not include many APOE e4 non-carriers, hence, only e4 allele carriers were included in the following analyses.

### Standard Protocol Approval and Participant Consent

This study was approved by the University of Illinois Institutional Review Board and, in accordance with the stated guidelines, all participants read and signed informed consent documents.

### Nutrient Biomarker Acquisition and Analysis

Fasting plasma was collected between 7:00 am and 12:00 noon Central Standard Time from January 2013 to May 2014. Gas chromatography equipped with a flame ionization detector quantified plasma fatty acid concentrations. Plasma lipids were measured with standard enzymatic methods (Bowman et al., 2011). The two fatty acids assessed in the current study include docosahexaeonic acid (DHA, 22:6n-3) and eicosapentaenoic acid (EPA, 20:5n-3), both of which are ω-3 polyunsaturated fatty acids. Participants were divided into low vs. high DHA and EPA levels separately for each nutrient according to a median split. Low/high group assignment for DHA and EPA was the same for 39 of the 40 participants; therefore we computed a composite O3PUFA score by taking the average of DHA and EPA and then divided participants into low vs. high O3PUFA levels based on the composite O3PUFA values. (Bowman et al., 2013; Lotrich et al., 2013; McNamara et al., 2013).

### APOE Genotyping

APOE genotyping was determined using PCR (e4 carrier, y/n). The presence of the Hardy–Weinberg equilibrium was tested using a chi-square goodness-of-fit test (Hardy, 1908).

### Neuropsychological Tests

Executive function was measured by the Delis-Kaplan Executive Function System (D-KEFS) Trail Making Test (Delis et al., 2006). This assessment yields a measure of executive function that can be isolated from underlying skills, including visual scanning, number sequencing, letter sequencing, and motor speed. In this task, participants alternate between multiple task goals (either number or letter sequencing), which elicits a specific type of executive function known as cognitive flexibility. The reported results from the D-KEFS Trail Making Test assess cognitive flexibility while controlling for motor speed and therefore provide a measure of cognitive flexibility that is not confounded by motor skill, which may be an important consideration in an elderly cohort.

### Volumetric Brain MRI

Volumetric analysis was performed on data from a 3D high-resolution (0.9 mm isotropic) T1-weighted scan using MPRAGE acquisition. Cortical reconstruction was performed with the Freesurfer image analysis suite, which is documented and freely available for download online.^1^ The technical details of these procedures are described in prior publications (Dale and Sereno, 1993; Dale et al., 1999; Fischl et al., 1999a,b, 2001, 2002, 2004a,b; Fischl and Dale, 2000; Ségonne et al., 2004; Han et al., 2006; Jovicich et al., 2006; Reuter et al., 2010, 2012). All cortical reconstructions were manually checked for accuracy, as recommended by the software developers. This analysis focused on gray matter volumes in the PFC provided by freesurfer parcellation. These regions included the superior frontal cortex, rostral middle frontal cortex, the caudal middle frontal cortex, pars opercularis, pars triangularis, pars orbitalis, lateral orbitofrontal cortex, medial orbitofrontal cortex, precentral gyrus, paracentral gyrus, frontal pole, rostral anterior cingulate cortex, and caudal anterior cingulate cortex. In particular, the analysis sought to assess the specific and sensitive role of the rostral anterior cingulate, and exclude the involvement of other regions within the PFC.

### Covariates

Covariates were included according to previous association with cognitive decline. These included age (continuous), gender (man/woman), education (nominal, year, fixed levels), income (nominal, five fixed levels), body mass index (BMI), and depression status (y/n). Although all participants had received a diagnosis of no depression at enrollment, the SF-36 Health Survey (Ware et al., 1993) revealed two participants with symptoms consistent with depression and so, in accordance with other studies, this was considered in the analysis as a covariate. PFC gray matter volume (continuous) was also included as a covariate in mediation analyses to assess the relationship between specific regions within the PFC, O3PUFA levels, and cognitive flexibility. These covariates were included in each of the four steps of the mediation analysis.

### Statistical Analyses

All statistics were performed in MATLAB v8.3.0.532 software. The variables of interest included the following: O3PUFA levels in blood, gray matter volumes of regions within the PFC provided by freesurfer parcellation, and cognitive flexibility.

A formal mediation analysis was used in an effort to better understand the relationship between O3PUFA levels, gray matter volume of regions within the PFC, and cognitive flexibility using a four-step framework (Baron and Kenny, 1986). In each step of the mediation, a regression model was estimated and adjusted for all covariates listed above. The first step examined the relationship between O3PUFA levels and gray matter volumes in the PFC (path a). The second step examined the relationship between O3PUFA levels and cognitive flexibility (path c). The third step tested the direct pathway of mediation by including both gray matter volumes in the PFC and O3PUFA levels as independent variables, and cognitive flexibility as the dependent variable (paths b and c considered together). The fourth step tested the indirect pathway of mediation, namely the interaction between paths a and b, using the Sobel *z*-test (path c’). A perfect mediation was indicated if the first three steps showed statistically significant relationships. A partial mediation was indicated if the first two steps showed statistically significant relationships, the third step showed non-significant relationships, and the Sobel *z*-test indicated a significant interaction between paths a and b.

## Results

### Participant Characteristics

Participants had a mean age of 68.80 years and 72.5 percent of participants were females. Mean O3PUFA level in the low O3PUFA group was 102.30 nmol/mL and mean O3PUFA level in the high O3PUFA group was 216.00 nmol/mL. The mean MMSE score was 28.83 and the D-KEFS Trail Making Test cognitive flexibility score was 10.30 (Table 1). APOE genotype distribution did not deviate from Hardy-Weinberg equilibrium (*χ*^2^ = 2.93, *p* > 0.05).

**Table 1:** **Characteristics of study population**.

**Demographics**	**Total *n* = 40**
Age (years)	68.80 ± 2.76
Female, n (%)	29 (72.5)
Education, n (%)	5 (12.5) high school degree
	7 (17.5) some college
	28 (70.0) college degree
Income, n (%)	2 (5.0) $15,000–$25,000
	8 (20.0) $25,000–$50,000
	9 (22.5) $50,000–$75,000
	8 (20.0) $75,000–$100,000
	13 (32.5) >$100,000
Depression, n (%)	38 (95.0) no
	2 (5.0) yes
**Plasma nutrients**	**(nmol/mL ± std)**
Low O3PUFA	102.30 ± 25.76
High O3PUFA	216.00 ± 48.43
**Psychometrics**	**(mean ± std)**
MMSE	28.83 ± 1.11
Cognitive flexibility score	10.30 ± 3.12
**Volumetric MRI (gray matter volume)**	**(mm^3^ ± std)**
Left frontal lobe	27400 ± 3240
Right frontal lobe	27197 ± 2984
Left superior frontal	19774 ± 2137
Right superior frontal	19245 ± 2203
Left rostral middle frontal	13769 ± 1756
Right rostral middle frontal	14367 ± 1959
Left caudal middle frontal	5764 ± 913
Right caudal middle frontal	5268 ± 953
Left pars opercularis	4291 ± 553
Right pars opercularis	3547 ± 542
Left pars triangularis	3183 ± 382
Right pars triangularis	3782 ± 560
Left pars orbitalis	2027 ± 262
Right pars orbitalis	2417 ± 375
Left lateral orbitofrontal	6744 ± 891
Right lateral orbitofrontal	6612 ± 844
Left medial orbitofrontal	4674 ± 710
Right medial orbitofrontal	4746 ± 697
Left precentral gyrus	12242 ± 1477
Right precentral gyrus	12044 ± 1526
Left paracentral gyrus	3105 ± 488
Right paracentral gyrus	3515 ± 430
Left frontal pole	815 ± 185
Right frontal pole	1123 ± 264
Left rostral anterior cingulate	2496 ± 475
Right rostral anterior cingulate	2098 ± 426
Left caudal anterior cingulate	1757 ± 353
Right caudal anterior cingulate	1864 ± 335

### Plasma O3PUFA and Cognitive Flexibility, Mediated by Rostral Anterior Cingulate Structure

The mediation analyses indicated that out of all regions within the PFC, gray matter volume of only the left rostral anterior cingulate partially mediates the relationship between O3PUFA blood levels and cognitive flexibility. Each relationship within the mediation is described below in a stepwise fashion.

**Figure 1:**
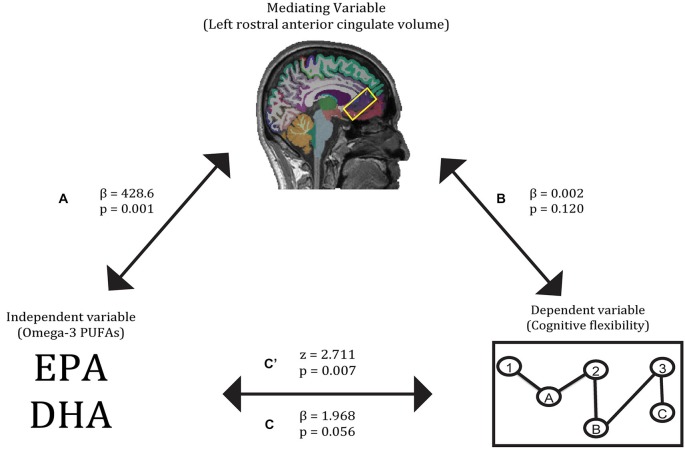
**Gray matter volume of the left rostral anterior cingulate partially mediates the relationship between O3PUFA blood levels and cognitive flexibility.** Paths A, B, C, and C’ refer to the four pathways tested in the mediation analysis.

First, O3PUFA levels associated with higher volume of the left rostral anterior cingulate cortex (*β* = 428.6, *p* = 0.001), with O3PUFA levels explaining 68.7% of the variation in cortical volume (a). Second, O3PUFA levels marginally associated with better cognitive flexibility (*β* = 1.968, *p* = 0.056), with O3PUFA levels explaining 53.4% of the variance in cognitive flexibility (c). Third, when adding both O3PUFA levels and left rostral anterior cingulate volume as independent variables, neither independent variable was a significant predictor of cognitive flexibility (*β* = 0.002, *p* = 0.120 for left rostral anterior cingulate volume; *β* = 1.433, *p* = 0.209 for O3PUFA levels), indicating the lack of a perfect mediation. Finally, the Sobel *z*-test showed a significant interaction between paths a and b (*z* = 2.711, *p* = 0.007), indicating that the volume of this brain region is a partial mediator of the relationship between O3PUFA levels and cognitive flexibility (Figure 1).

## Discussion

This study found that volume of the left rostral anterior cingulate mediates the relationship between O3PUFA levels and cognitive flexibility. This is the first report of a specific and sensitive volumetric mediation between O3PUFA levels and a particular component of the executive functions, and is shown in an at-risk population of cognitively intact older adults. The individual relationships reported within the mediation, including those between O3PUFA levels and cognitive flexibility, between O3PUFA levels and left rostral anterior cingulate, and between left rostral anterior cingulate and cognitive flexibility, are substantiated by prior findings described below.

The first relationship demonstrated a positive association between higher O3PUFA blood levels and larger volume in the rostral anterior cingulate cortex of the left hemisphere (Figure 1, path A). Past studies have also linked higher O3PUFA blood levels to superior anterior cingulate structure and function (Conklin et al., 2007; McNamara et al., 2013; Bauer et al., 2014). The second relationship revealed higher O3PUFA levels were associated with better cognitive flexibility (Figure 1, path C). In past work, higher O3PUFA intake has been linked to better performance on tasks of cognitive flexibility (Beydoun et al., 2007; Bowman et al., 2013; Witte et al., 2013). The indirect pathway of mediation indicated a mediatory effect of left rostral anterior cingulate gray matter volume on the relationship between O3PUFA blood levels and cognitive flexibility (Figure 1, path C). Higher gray matter volumes in the anterior cingulate cortex have been linked to higher O3PUFA intake as well as superior cognitive flexibility, as evidenced by measures of task switching (Conklin et al., 2007; Huster et al., 2009; Nee et al., 2011; Witte et al., 2013; Raji et al., 2014). The unilateral nature of this mediator is substantiated by prior work implicating the vulnerability of regions within the left hemisphere to Alzheimer’s disease-related degeneration and associated cognitive impairments (Chételat et al., 2005; Querbes et al., 2009; Risacher et al., 2010; Mosconi et al., 2014).

Cognitive aging literature indicates that there are two major postulated routes through which O3PUFAs might impact executive functions, namely the vascular and non-vascular hypotheses. In reporting a volumetric mediation between O3PUFA levels and cognitive flexibility, our study is the first to provide evidence for the non-vascular hypothesis within a formal mediation framework. While the vascular hypothesis suggests that reducing blood pressure, lowering risk of thrombosis, reducing inflammation, and lowering serum triglyceride levels can prevent deterioration of the brain (Keli et al., 1994; Calder, 2004; Kelley et al., 2008), the non-vascular hypothesis states that cortical and subcortical volumes show reduction in healthy aging due to mechanisms not directly related to changes in vasculature. These effects are especially robust in the frontal cortex, and prevention or reduction of this atrophy can prevent cognitive decline (Salat et al., 2004). The non-uniform deterioration of both executive functions and PFC structure associated with aging support the notion that O3PUFAs may slow age effects on specific aspects of the executive functions and in particular regions within the PFC (MacPherson et al., 2002). Possible non-vascular mechanisms that may underlie this volumetric mediation include enhanced membrane and membrane-bound protein function, reduced amyloid-β production, reduced neuroinflammation and oxidative damage, increased levels of brain derived neurotrophic factor, and reduced excitotoxic omega-6 levels (Cole and Frautschy, 2010).

The partial nature of this mediation is supported by the specificity of this analysis, which honed in on one component of the executive functions and one region within the PFC. Brain aging is a dynamic, heterogeneous, and multi-faceted process (Koepsell and Monsell, 2012), and it is unlikely that the volume of a specific region within the PFC would completely and exclusively mediate the relationship between this vital nutrient for healthy aging and such an important cognitive function in aging. Rather than claiming that the rostral anterior cingulate is the sole mediator of the relationship between O3PUFAs and cognitive flexibility, this study is one of the first to begin defining specific and sensitive mediatory factors between nutrition and cognition.

The strength of this study includes the use of blood biomarkers to measure nutrition, which provided a more reliable assessment of dietary intake than that of food frequency questionnaires. The potential limitations of this study include the cross-sectional design, relatively small sample size, and inability to assess this relationship in both carriers and non-carriers of the APOE e4 allele. Future longitudinal studies with larger samples size are needed to confirm these results.

## Conflict of Interest Statement

The authors declare that the research was conducted in the absence of any commercial or financial relationships that could be construed as a potential conflict of interest.

## Footnotes

^1^http://surfer.nmr.mgh.harvard.edu/
